# Factors for Social Stressors Among Type 2 Diabetics Versus Non-Diabetics Using the Hamilton Depression Rating Scale

**DOI:** 10.7759/cureus.11861

**Published:** 2020-12-02

**Authors:** Saadia Y Raja, Uzma Ghori, Haider Ali Naqvi, Sadaf Aijaz, Adnan Anwar, Atif A Hashmi

**Affiliations:** 1 Internal Medicine, Ziauddin University, Karachi, PAK; 2 Psychiatry, Ziauddin University, Karachi, PAK; 3 Psychiatry, Northern Border University, Arar, SAU; 4 Physiology, Al-Tibri Medical College, Isra University, Karachi, PAK; 5 Pathology, Liaquat National Hospital and Medical College, Karachi, PAK

**Keywords:** social stressors, depression, anxiety, type ii diabetes, hamilton depression rating scale (ham-d)

## Abstract

Objective

This study aimed to evaluate different factors of social stressors among people with type II diabetes versus non-diabetics by using the Hamilton Depression Rating Scale (HAM-D).

Methodology

This case-control study was done for one year at Ziauddin University Hospital, Karachi, Pakistan. Diagnosed patients with type II diabetes between 25 and 60 years were included as cases and age-related healthy individuals as controls. Participants on any psychotropic medications, neurodegenerative disorders, or on chemotherapy were excluded. Hamilton Rating Scale for Depression (HRDS-17) was used for recording depressive symptoms. The Statistical Package for the Social Sciences (SPSS) version 26.0 (IBM Corp., Armonk, NY) was used for data analysis. The frequency was calculated for descriptive statistics and depression scores (HRDS-17). A significance level of 0.05 was considered.

Results

A total of 272 patients were divided into two groups, with 136 patients in the group with diabetes and 136 participants in the control group. The mean age among people with diabetes was 55.13±9.10 years and among non-diabetics was 43.25±12.97 years (p<0.001). The mean duration of illness in people with diabetes was 8.51±7.57 years and in non-diabetics, it was 6.73±4.42 years (p=0.018). The mean social rating in people with diabetes was 164.0±155.60 and in non-diabetics, it was 124.75±99.02 (p=0.014). Insomnia, both in the early and middle part of the night affecting work activities, hypochondriasis, and loss of weight, was significantly present among diabetics as compared to controls (p<0.05).

Conclusion

Most diabetics reported a significant effect on the quality of life based on social rating and health-care expenditure. They experienced depressive symptoms more frequently than non-diabetics. Insomnia in the middle of the night affected work and activities; hypochondriasis and weight loss were found to be substantially higher among patients with diabetes than in controls.

## Introduction

The ever-increasing incidences of stress, anxiety, and depression among patients of type II diabetes mellitus (type II DM) are alarming as compared with the general population [1]. Type II DM is termed as a chronic illness, which is hazardous to health all over the globe, resulting in a significant disorder of lifestyle having a potential short and long-term incidence of complications. Alternatively, depression is one of the most commonly reported psychological disorders [2]. Patients with type II DM have been known to develop depression, therefore leading to unfavorable or worse outcomes. Type II DM causes a substantial burden on both medical and economic forums. Between 2010 and 2030, an approximately 69% increase in type II DM among adults has been estimated while it is approximately 20% in developed countries [3-4]. Nevertheless, the risk factors of both type II DM and depression are modifiable even when they occur as a long-term disorder. Type II DM is the leading cause of death, affecting around 264 million people worldwide [5].

In Pakistan, the prevalence of type II DM in the urban population above the age of 25 years is approximately 6.8% for males and 5.1% for females; the rates are lower in the rural community, i.e., 5% in males and 4.8% in females [6]. The worldwide prevalence of depression, both in the developed and developing worlds, is estimated to be 10%. Simultaneously, this frequency is reported to be much higher in Pakistan, approximately 34%, including both urban and rural diabetic populations [7]. In the rural community of Pakistan, rates of depression were recorded at 14.7% among patients with diabetes. Type II DM is linked with depression, leading to deterioration in the quality of life and substantially affecting compliance to treatment of both type II DM and depression. Losing interest in maintaining one's health, as well as dietary regimen or exercise, is commonly observed among patients with depression [8].

Type II DM can predispose a person to stress and depression, lowering his/her quality of life in terms of maintaining good health. The fear of depression's occurrence among patients with type II DM leads to not only a functional and financial but also a psychological burden on the person as well as his/her family, leading to poor glycemic control, noncompliance with medication, and worsening adherence with diet, exercise, and self-care [9-10].

Proper guidelines for an appropriate approach toward patients with type II DM with stress or depression and those without stress or depression are still under consideration and a matter of debate. The condition is termed as stress linked to diabetes. Combining type II DM with psychiatric disorders, such as depression, substance abuse, and anxiety, with reduced quality of life, leads to higher chances of hyperglycemia. All these conditions lead to significant difficulties in regulating blood glucose [11-12]. Therefore, they make patients with type II DM susceptible to diabetic and psychiatric complications. Studies have reported that among diabetic patients with depression, the use of medicines is recommended along with physical activity for increasing treatment effectiveness, as it leads to the release of B-endorphins and cerebral neurotransmitters. In turn, this decreases levels of stress and anxiety, which results in an improvement in the quality of life of these patients [13-14].

Therefore, this study aimed to evaluate the different factors of social stressors among people with type II diabetes as compared to non-diabetics by using the Hamilton Depression Rating Scale (HAM-D).

## Materials and methods

This case-control study was conducted at Ziauddin University Hospital, Karachi, Pakistan. The sampling technique was consecutive and the duration of the study was six months from January 2020 until May 2020. Diagnosed patients with type II diabetes between the ages of 25 and 60 years, having documented glycated hemoglobin (HbA1c) levels >6.5% or two reported random blood glucose levels of more than 200 mg/dl and on oral hypoglycemic drug/insulin treatment for a minimum of six months were included as the cases and age-related healthy individuals were included as the controls, with 136 subjects in each group. Subjects that fulfilled the inclusion criteria were included after giving written and informed consent. Type II diabetics patients on any psychotropic medications (antipsychotics and antidepressants), having neurodegenerative disorders such as Alzheimer's and multi-infarct dementia that can interfere with cognitive assessment, patients on chemotherapy due to malignancy, on drugs such as cannabinoids or opioids, alcohol users, and patients having any thyroid-related problems were excluded from the study. The Hamilton Rating Scale for Depression (HRDS-17) was used for recording depressive symptoms since it consists of a more significant number of somatic symptoms and less affective or cognitive symptoms.

Information related to participants was kept confidential and the study was conducted after taking approval from the ethical review committee of Ziauddin University. Their names and identities were not recorded. Each individual was given a unique identification number. Data analysis was performed using the Statistical Package for Social Sciences (Version 26.0, IBM Corp., Armonk, NY). Descriptive statistics were used for variables such as age, gender, duration of illness, HbA1c, health-care expenditure, social rating, total scores, and depression scores (HRDS-17). The chi-square test and t-test were used to assess the difference. A significance level of 0.05 was considered.

## Results

A total of 272 patients were divided into two groups with 136 patients in the group with diabetes and 136 participants in the control group, the mean age of the group with diabetes was 55.13±9.10 years, and in the non-diabetic group, it was 43.25±12.97 years with a substantial difference between the groups (p<0.001). The mean duration of illness in the group with diabetes was 8.51±7.57 years. The mean HbA1c in the group with diabetes was 6.91±1.40, and in the non-diabetic group, it was 5.78±0.87 with a substantial difference between the groups (p<0.001). The mean health-care expenditure in the group with diabetes was Rs. 7180.88±5356.20/year and in the non-diabetic group was Rs. 5888.23±4286.18/year, with a significant difference between the groups (p=0.029). The mean social rating in the group with diabetes was 164.0±155.60, and in the non-diabetic group, it was 124.75±99.02, with a significant difference between the groups (p=0.014) (Table 1).

**Table 1:** Baseline demographics of diabetic versus non-diabetic group PKR: Pakistani rupees

Variable		Diabetic (Mean ± SD)	Non-diabetic (Mean ± SD)	P-values
Age		55.13±9.10	43.25±12.97	<0.001
Gender	Male	55(40.4%)	67(49.3%)	0.088
	Female	81(59.6%)	114(83.8%)	
Duration of Illness (years)		8.51±7.57	6.73±4.42	0.018
HbA1c		6.91±1.40	5.78±0.87	<0.001
Health Care Expenditure (PKR/year)		7180.88±5356.20	5888.23±4286.18	0.029
Social Rating		164.0±155.60	124.75±99.02	0.014
Total Score		10.44±7.87	8.77±7.11	0.069

No insomnia in the middle of the night was reported in 84 (61.8%), agitation and disturbed sleep during the night in 48(35.3%), and waking during the night (except for purposes of voiding) in four (2.9%) in the group with diabetes (p=0.05). Hypochondriasis was not seen in 84 (61.8%), self-absorption (bodily) in 31 (22.8%), preoccupation with health in 12 (8.8%), and frequent complaints in 9 (6.6%) in the group with diabetes (p=0.004). Loss of weight (according to the weekly patient measurement) was not reported as weight loss in 87 (64.0%), probable weight loss associated with present illness in 22 (16.2%), definite (according to the patient) weight loss in seven (5.1%), and not assessed in 20 (14.7%) in the group with diabetes (p=0.015) (Table 2).

**Table 2:** Hamilton Depression Rating Scale (HAM-D) between diabetic and non-diabetic groups

Variable			Diabetic			Non-diabetic		P-value	
Depressed Mood (sadness, hopeless, helpless, worthless)	Absent		76(55.9%)			80(58.8%)		0.889	
	These emotion states indicated only on questioning		27(19.9%)			30(22.1%)			
	These feeling states spontaneously reported verbally		17(12.5%)			14(10.3%)			
	Communicates feeling states non-verbally, i.e. through facial expression, posture, voice and tendency to weep		9(6.6%)			7(5.1%)			
	Patient reports virtually only these feeling states in his/her spontaneous verbal and non-verbal communication		7(5.1%)			5(3.7%)			
Feelings of Guilt	Absent		98(72.1%)			91(66.9%)		0.140	
	Self reproach, feels he/she has let people down		14(10.3%)			22(16.2%)			
	Ideas of guilt or rumination over past errors or sinful deeds		13(9.6%)			19(14.0%)			
	Present illness is a punishment. Delusions of guilt		10(7.4%)			3(2.2%)			
	Hears accusatory or denunciatory voices and/or experiences threatening visual hallucinations		1(0.7%)			1(0.7%)			
Suicide	Absent		122(89.7%)			125(91.9%)		0.222	
	Feels life to be useless to live		8(5.9%)			8(5.9%)			
	Wishes he/she were dead or any thoughts of possible death to self		5(3.7%)			1(0.7%)			
	Ideas or gestures of suicide		0(0.0%)			2(1.5%)			
	Attempts at suicide		1(0.7%)			0(0.0%)			
Insomnia: Early in the Night	No difficulty falling asleep		72(52.9%)			90(66.2%)		0.056	
	Complains of occasional difficulty falling asleep, i.e. more than 1/2 hour		52(38.2%)			34(25.0%)			
	Complains of nightly difficulty falling asleep		12(8.8%)			12(8.8%)			
Insomnia: Middle of the Night	No difficulty		84(61.8%)			97(71.3%)		0.052	
	Patient reports for agitation and disturbed during the night		48(35.3%)			31(22.8%)			
	Waking during the night-any getting out of bed rates 2 (except for purposes of voiding)		4(2.9%)			8(5.9%)			
Variable				Diabetic	Non-diabetic		P-value		
Insomnia: Early Hours of the Morning		No difficulty		75(55.1%)	82(60.3%)		0.320		
		Waking in early hours of the morning but goes back to sleep		36(26.5%)	39(28.7%)				
		Unable to fall asleep again if he/she gets out of bed		25(18.4%)	15(11.0%)				
Work and Activities		No difficulty		63(46.3%)	84(61.8%)		0.007		
		Thoughts and feelings of incapacity, fatigue or weakness related to activities, work or hobbies		56(41.2%)	35(25.7%)				
		Loss of interest in activity, hobbies or work-either directly reported by the patient or indirect in listlessness		10(7.4%)	9(6.6%)				
		Decrease in actual time spent in activities or decrease in productivity		3(2.2%)	8(5.9%)				
		Stopped working because of present illness		4(2.9%)	0(0.0%)				
Retardation (slowness of thought and speech, impaired ability to concentrate, decreased motor activity)		Normal speech and thought		102(75.0)	110(80.9%)		0.488		
		Slight retardation during the interview		26(19.1%)	22(16.2%)				
		Obvious retardation during the interview		4(2.9%)	1(0.7%)				
		Interview difficult		3(2.2%)	3(2.2%)				
		Complete stupor		1(0.7%)	0(0.0%)				
Agitation		None		83(61.0%)	87(64.0%)		0.231		
		Fidgetiness		42(30.9%)	34(25.0%)				
		Playing with hands, hair, etc		5(3.7%)	10(7.4%)				
		Moving about, can’t sit still		6(4.4%)	3(2.2%)				
		Hand wringing, nail biting, hair-pulling, biting of lips		0(0.0%)	2(1.5%)				
Anxiety psychic		No difficulty		69(50.7%)	68(50.0%)		0.713		
		Subjective tension and irritability		35(25.7%)	38(27.9%)				
		Worrying about minor matters		22(16.2%)	25(18.4%)				
		Apprehensive attitude apparent in face or speech		7(5.1%)	3(2.2%)				
		Fears expressed without questioning		3(2.2%)	2(1.5%)				
Anxiety Somatic		Absent		69(50.7%)	79(58.1%)		0.411		
		Mild		42(30.9%)	41(30.1%)				
		Moderate		23(16.9%)	14(10.3%)				
		Severe		2(1.5%)	2(1.5%)				
		Incapacitating		0(0.0%)	0(0.0%)				
Somatic Symptoms Gastro-Intestinal		None		97(71.3%)	92(67.6%)		0.272		
		Loss of appetite but eating without staff encouragement. Heavy feelings in abdomen		31(22.8%)	40(29.4%)				
		Difficulty eating without staff urging. Requests or requires laxatives or medication for bowels or medication for gastro-intestinal symptoms		8(5.9%)	4(2.9%)				
General Somatic Symptoms		None		74(54.4%)	81(59.6%)		0.362		
		Heaviness in limbs or head. Backaches, headaches, muscle aches. Loss of energy and fatigability		61(44.9%)	52(38.2%)				
		Any clear-cut symptom		1(0.7%)	3(2.2%)				
Genital Symptoms		Absent		102(75.0%)	111(81.6%)		0.414		
		Mild		31(22.8%)	23(16.9%)				
		Severe		3(2.2%)	2(1.5%)				
Hypochondriasis		Not present		84(61.8%)	108(79.4%)		0.004		
		Self-absorption (bodily)		31(22.8%)	14(10.3%)				
		Preoccupation with health		12(8.8%)	4(2.9%)				
		Frequent complaints, requests		9(6.6%)	10(7.4%)				
Loss of Weight (rate either a or b) (A: According to the patient)		Absence of loss of weight		87(64.0%)	107(78.7%)		0.015		
		possible weight loss related with present illness		22(16.2%)	7(5.1%)				
		Definite (according to patient) weight loss		7(5.1%)	5(3.7%)				
		Not assessed		20(14.7%)	17(12.5%)				
(B: According to weekly patient: measurement)		Less than 1 lb weight loss in week		84(61.8%)	96(70.6%)		0.493		
		Greater than 1 lb weight loss in a week		5(3.7%)	4(2.9%)				
		Greater than 2 lb weight loss in a week		1(0.7%)	1(0.7%)				
		Not assessed		46(33.8%)	35(25.7%)				
Insight		Acknowledges being depressed and ill		73(53.7%)	73(53.7%)		0.321		
		Acknowledges illness but attributes cause to bad food, climate, overwork, etc.		29(21.3%)	20(14.7%)				
		Denies being ill at all		34(25.0%)	42(31.6%)				

## Discussion

According to the present study results, anxiety and depressive symptoms were observed higher among diabetics than controls. Likewise, in the analysis of studies regarding anxiety/depression, a higher prevalence has been reported among people with diabetes than controls [15-17]. In this regard, a study observed that a high probability existed in patients with diabetes for developing anxiety or depression [18]. Even though the association of anxiety and depression with diabetes mellitus has long been stated, yet the frequency of symptoms is rapidly increasing worldwide [19-21]. As a result, it is necessary to observe the presence of such symptoms among people with diabetes, especially to improve compliance to treatment, which will result in a positive impact on diabetic control [22-23]. For this purpose, a study conducted to determine the frequency of anxiety and depression among 820 patients with type II diabetes using the HAM-D discovered that 48.27% and 55.1% of the patients with diabetes in the study were suffering from depression and anxiety, respectively. The main reasons reported for the cause of anxiety issues were attributed to occupation and complications in diabetes while glucose levels and difficulties in diabetes were linked to depression. A highly significant factor for both anxiety and depression was diabetic complications [24].

In yet another study evaluating the relationship between major depression and the glucose control index among type II diabetics, from 134 patients, using HAM-D, a significantly higher depressive score was reported among patients with diabetes having hypertension (p=0.001), asthma (p-0.01), and on insulin treatment (p=0.005) [25]. Similar findings were reported in our study as well. However, in contrast to our research, the above research reported an insignificant association between the level of depressive symptoms and HbA1c among type II diabetes patients.

Another study determining the prevalence of psychiatric morbidity among diabetic patients using standardized HAM-D scales for depression and anxiety reported that approximately 84% of patients had comorbid depression in which females were found to have a higher percentage of anxiety and depression. Besides, the severity of depression was also elevated among females. Genital symptoms were reported higher among males, whereas somatic symptoms were common among females [26]. However, in our study, we reported the frequencies among the diabetic and control groups, including males and females, without analyzing them separately. Overall, an insignificant association was reported in terms of depression in type II diabetics in males and females. Another study determined the frequency of depression among type II diabetics and reported that 38.75% of patients suffered from depression, among which 48.33% were found to have severe depression. However, an insignificant association was reported between depression in type II diabetics with sociodemographic factors like age (p = 0.92), gender (p = 0.25), education (p = 94), and marital status (p = 0.064) [27]. Similarly, in another study, using the HAM-D scale, 34% of type II diabetics were found to have depression and anxiety. The above studies reported that diabetic patients that were depressed were severely affected in their daily routine activities [28]. Likewise in our study as well, depressed patient's daily activities were severely affected (p=0.007). In accordance with our study findings, yet another study reported around half of type II diabetics to have depression with significant deficits in cognitive function, affected sleep, and loss of weight [29].

Limitations of the study

Although the study evaluated several aspects of the type of diabetes associated with depression and anxiety and its effect on patients' quality of life, it was not immune to selection and recall biases. In addition to selection and recall bias, the study was conducted in a single center, thereby being performed on patients belonging to a specific socio-economic class. Socio-economic status and family support were also undetermined in the study. Another limitation of our study was the small sample size, therefore, we recommend large-scale prospective studies in our population to assess the impact of depression and anxiety in diabetic patients.

## Conclusions

According to the results of the study, most of the patients with diabetes in the study reported a significant effect on their quality of life based on social rating and health-care expenditure, and they experienced depressive symptoms more frequently than non-diabetics. However, in terms of social stressors and depressive symptoms/illness, most of the stressors did not report significant differences between patients with diabetes and the controls. Insomnia in the middle of the night affected their work and activities; hypochondriasis and loss of weight were found to be substantially higher among patients with diabetes as compared with controls.
